# Detection of Favorable QTL Alleles and Candidate Genes for Lint Percentage by GWAS in Chinese Upland Cotton

**DOI:** 10.3389/fpls.2016.01576

**Published:** 2016-10-21

**Authors:** Junji Su, Shuli Fan, Libei Li, Hengling Wei, Caixiang Wang, Hantao Wang, Meizhen Song, Chi Zhang, Lijiao Gu, Shuqi Zhao, Guangzhi Mao, Chengshe Wang, Chaoyou Pang, Shuxun Yu

**Affiliations:** ^1^State Key Laboratory of Cotton Biology, Institute of Cotton Research of CAASAnyang, China; ^2^Department of Plant Sciences, College of Agronomy, Northwest A&F UniversityYangling, China

**Keywords:** upland cotton, lint percentage, genome-wide association study, favorable haplotypes, candidate genes

## Abstract

Improving cotton yield is a major breeding goal for Chinese upland cotton. Lint percentage is an important yield component and a critical economic index for cotton cultivars, and raising the lint percentage has a close relationship to improving cotton lint yield. To investigate the genetic architecture of lint percentage, a diversity panel consisting of 355 upland cotton accessions was grown, and the lint percentage was measured in four different environments. Genotyping was performed with specific-locus amplified fragment sequencing (SLAF-seq). Twelve single-nucleotide polymorphisms (SNPs) associated with lint percentage were detected via a genome-wide association study (GWAS), in which five SNP loci distributed on chromosomes A_t_3 (A02) and A_t_4 (A08) and contained two major-effect QTLs, which were detected in the best linear unbiased predictions (BLUPs) and in more than three environments simultaneously. Furthermore, favorable haplotypes (FHs) of two major-effect QTLs and 47 putative candidate genes in the two linkage disequilibrium (LD) blocks of these associated loci were identified. The expression levels of these putative candidate genes were estimated using RNA-seq data from ten upland cotton tissues. We found that *Gh_A02G1268* was very highly expressed during the early fiber development stage, whereas the gene was poorly expressed in the seed. These results implied that *Gh_A02G1268* may determine the lint percentage by regulating seed and fiber development. The favorable QTL alleles and candidate genes for lint percentage identified in this study will have high potential for improving lint yield in future Chinese cotton breeding programs.

## Introduction

Upland cotton (*Gossypium hirsutum* L.; AADD, 2n = 4x = 52) is the most important natural textile fiber source worldwide, accounting for approximately 95% of the world's cotton production (Chen et al., 2007). Although fiber quality traits, which are of upmost importance to cotton breeding programs, have dominated these studies in cotton (Rong et al., 2007; Said et al., 2013), improving cotton yield is still a primary goal in Chinese cotton breeding. Lint percentage is an important yield component and a critical economic index for cotton cultivars, and many studies have showed that raising the lint percentage is closely related to cotton lint yield improvement (Culp and Harrell, 1975; Li et al., 2009; Zeng and Meredith, 2009). However, the genetic and molecular mechanisms underlying variations in lint percentage in upland cotton remain poorly understood.

Quantitative trait locus (QTL) mapping has been widely used to dissect the genetic changes for cotton complex traits, such as fiber quality and yield component traits (Rong et al., 2007; Said et al., 2013, 2015). Over the past two decades, a number of QTLs for lint percentage have been identified through biparental linkage mapping in upland cotton (Zhang et al., 2005; Abdurakhmonov et al., 2007; Shen et al., 2007; Wan et al., 2007; Liu et al., 2012, 2015; Yu et al., 2013a,b; Wang et al., 2014). Association mapping as an alternative for detecting QTLs has been used widely in QTL mapping for important economic traits in cotton, such as fiber quality traits (Abdurakhmonov et al., 2008; Zhang et al., 2013; Nie et al., 2016), yield and its components (Mei et al., 2013; Qin et al., 2015), and resistance traits (Zhao et al., 2014). However, reports of association analysis for lint percentage in upland cotton are still rare. Only a small number of simple sequence repeat (SSR) and amplified fragment length polymorphism (AFLP) markers associated with lint percentage have been identified through association mapping (Mei et al., 2013; Badigannavar and Myers, 2015; Qin et al., 2015). In recent years, genome-wide association studies (GWASs) have become a higher-resolution and more cost-effective tool for detecting important QTLs or genes associated with complex traits compared with linkage mapping. Although, GWASs have been widely performed for a large number of single-nucleotide polymorphisms (SNPs) in *Arabidopsis* (Zhao et al., 2007; Atwell et al., 2010), maize (Kump et al., 2011) and rice (Huang et al., 2010; Zhao et al., 2011), they have seldom been used for large quantities of SNPs in cotton.

Upland cotton is an allopolyploid species with a complex genome structure due to high homology between its A- and D-subgenomes. Although this situation imposes a huge challenge for the high-throughput discovery of high-quality SNPs for GWAS, next-generation sequencing technologies, such as genotyping-by-sequencing (GBS), restriction site-associated DNA sequencing (RAD-seq) and specific-locus amplified fragment sequencing (SLAF-seq), have provided opportunities to identify required marker coverage in upland cotton (Gore et al., 2014; Chen et al., 2015; Wang H. et al., 2015; Wang Y. et al., 2015; Zhang et al., 2016). To better understand the genetic variations of lint percentage at a natural population level, a diversity panel consisting of 355 upland cotton accessions was genotyped by SLAF-sequencing. The lint percentage was measured in four different environments. A GWAS was performed to identify SNP loci or QTL regions associated with the lint percentage trait in upland cotton. The candidate genes that control lint percentage were further predicted by RNA-seq analysis in major-effect QTL regions. These results will lay the foundation for lint percentage improvement through marker-assisted breeding.

## Materials and methods

### Plant materials

A diversity panel consisting of 355 representative upland cotton accessions (Supplementary Table S1) obtained from cotton germplasm collections in our laboratory and from the low-temperature germplasm genebank of the Cotton Research Institute, Chinese Academy of Agricultural Sciences (CRI-CAAS), was examined. The population consisted of 331 cultivars or accessions developed in China and 24 introduced from abroad. These accessions were divided into the following five groups according to their ecological areas: (1) YR group (162 accessions from the Yellow River region in China), (2) YZR group (51 accessions from the Yangtze River region in China), (3) NW group (98 accessions from the northwest inland region in China), (4) LN group (20 accessions from the Liaoning province in China) and (5) foreign group (20 accessions from the United States of America and 4 accessions from central Asia countries).

### Field experiments and phenotyping

All of the 355 upland cotton accessions were planted at Anyang, Henan, China (36⋅ 08′ N, 114⋅ 48′ E) in 2014 and 2015 (designated AY-14 and AY-15, respectively) and at Shihezi (SHZ), Xinjiang, China (44⋅ 31′ N, 86⋅ 01′ E) in 2014 and 2015 (designated SHZ-14 and SHZ-15, respectively). The field experiments followed a randomized complete block design with three replications. At Anyang, Henan, each accession was grown in a single-row plot (5.00 m long and 0.80 m row wide) with 18–23 plants, while at Shihezi (SHZ), Xinjiang, each accession was grown in a plot with 30–40 plants in two rows, with 0.10 m between the plants in each row and 0.45 m between the rows. The trial management followed standard breeding field practices. The fully opened random 20 cotton middle bolls for each accession were manually harvested annually in September. The lint percentage (LP, %) of each harvested sample was evaluated. The correlation coefficients for the lint percentage between environments were calculated and the analysis of variance (ANOVA) was conducted using R software. The broad-sense heritability of lint percentage was calculated using the R software package “lem4.”

### SNP genotyping

Genomic DNA from all of the accessions was extracted from young leaf tissue using a modified cetyl trimethyl ammonium bromide (CTAB) method (Paterson et al., 1993). SNP genotyping of the association panel was performed using a SLAF-seq approach (Sun et al., 2013). Two restriction enzymes (*Rsa I* and *Hae III*, New England Biolabs, NEB, USA) were used for library preparation. Paired-end sequencing (125 bp at each end) was performed on an Illumina HiSeq 2500 system (Illumina, Inc., San Diego, CA, USA) according to the manufacturer's recommendations. The GATK and Samtools packages were used for SNP calling. BWA software was used to map the raw paired-end reads onto the reference genome (*Gossypium hirsutum* v 1.0) (Li et al., 2015). SNPs with a missing rate < 10% and a minor allele frequency (MAF) ≤ 0.05 were excluded. A total of 81,675 SNP markers were identified and used for the subsequent analysis.

### Genome-wide association study

The best linear unbiased predictions (BLUPs) for lint percentage across the four environments were estimated using R software. The BLUP values for the four environments and the phenotypic values for each environment's lint percentage were used for GWAS. A mixed linear model (MLM) was used to calculate the associations in all of the analyses by incorporating both principal components (PCs) and kinship data (Lipka et al., 2012). The suggestive and significant *P* thresholds were 6.12E–06 (*P* = 0.5/n, where *n* = the number of markers used; −log_10_ (0.5/81,675) = 5.21) and 6.12E–07 (*P* = 0.05/n, where *n* = the number of markers used; −log_10_ (0.05/81,675) = 6.21), respectively, for the entire population (Holm, 1979; Mao et al., 2015). Manhattan plots were performed using the R software package “CMplot.”

### Haplotype analysis

The phenotypic value of each haplotype was estimated by averaging the phenotypic values over the accessions with each type of SNP locus associated with a specific target trait. The favorable haplotypes (FHs) were subsequently identified according to the breeding objectives of target trait. Box plots for the relative phenotypic values were performed using R software.

### Candidate gene annotation and prediction

Linkage disequilibrium (LD) blocks containing SNP loci associated with target traits were generated using the R software package “LDheatmap” (Shin et al., 2013). LD blocks harboring significantly associated SNPs were then defined as the candidate genome regions. The genes distributed in these regions were collected. The gene annotation closest to the *Arabidopsis* homolog was obtained from the *Arabidopsis* information resource (http://www.arabidopsis.org/). Transcriptome sequencing data (SRA: PRJNA248163) from 10 upland cotton (TM-1) tissues (including root, stem, leaf, torus, seed, cotyledon, fiber-5 DPA (days post-anthesis), fiber-10 DPA, fiber-20 DPA and fiber-25 DPA) were available on the EBI website (http://www.ebi.ac.uk/). Expression analysis of the high-throughput mRNA sequencing was performed using TopHat and Cufflinks software (Trapnell et al., 2012). Heat maps of the putative candidate gene expression patterns were created using the R package “pheatmap.”

## Results

### Phenotypic variation of lint percentage

**Figure 1:**
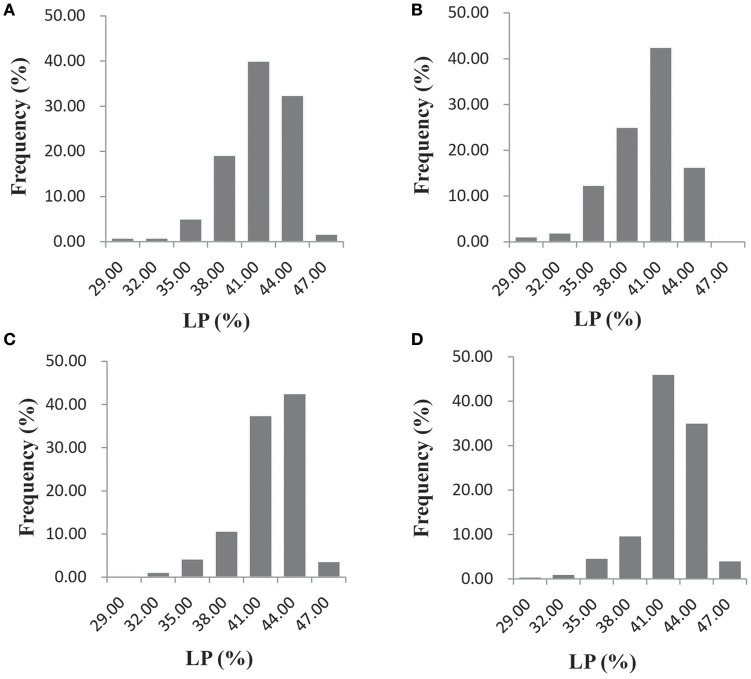
**Frequency distribution of the lint percentage (LP, %) in the four environments (AY-14, AY-15, SHZ-14, and SHZ-15) among the 355 upland cotton accessions. (A)** AY-14, **(B)** AY-15, **(C)** SHZ-14, and **(D)** SHZ-15.

Significant variation was observed for lint percentage among the 355 upland cotton accessions, ranging from 26.58 to 48.13%, with an average of 41.03%. In the AY-14, AY-15, SHZ-14, and SHZ-15 environments, the natural population exhibited average lint percentage values (±SD) of 41.05 ± 2.87, 39.70 ± 3.18, 41.78 ± 2.78, and 41.58 ± 2.64%, respectively (Figure 1). The ANOVA showed that the genotype (G), environment (E) and genotype × environment (G × E) interactions have significant effects on the lint percentage (*P* < 0.001, Supplementary Table S2). The phenotypic trait also showed significant differences (*P* < 0.01) among the four environments (Supplementary Table S2), suggesting that the lint percentage was significantly influenced by the environment. However, significant positive correlations (*P* < 0.001) between the genotypes across the four environments were observed, with correlation coefficients ranging from 0.7462 to 0.8620 (Supplementary Table S3). Additionally, the broad-sense heritability of lint percentage was as high as 69.72% (Supplementary Table S2). The results indicated that the stability of the lint percentage was high, even though significant G × E was observed.

### Genome-wide association studies (GWASs)

**Figure 2:**
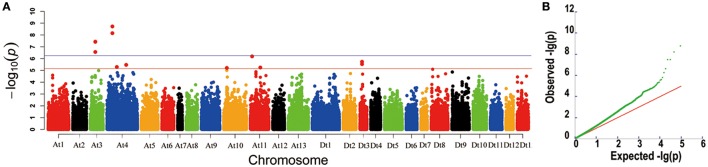
**Genome-wide association study for lint percentage. (A)** Manhattan plots of the mixed linear model (MLM) for the best linear unbiased prediction (BLUP) across the four environments of lint percentage. Each dot represents a SNP. The horizontal full red and blue lines indicate the Bonferroni-corrected significance thresholds at −log_10_(*P*) = 5.21 and −log_10_(*P*) = 6.21, respectively. **(B)** A quantile-quantile (QQ) plot of MLM for lint percentage.

In our previous studies, 355 upland cotton accessions were grouped into two major subpopulations by principal component analysis (PCA) and clustering analysis, and LD was estimated as *r*^2^ (the squared Pearson correlation coefficient) between all pairs of SNP markers; the approximated LD decay distance was 100 kb (Su et al., 2016). In this study, GWASs were conducted for lint percentage using the BLUPs across four environments and an individual environment in an MLM. The threshold of −log_10_ (*P*) > 5.21 was also derived from the quantile-quantile (QQ) plots because most of the upward deviation from the linear line occurred around −log_10_ (*P*) = 5.21, which presumably indicates true positives (Figure 2B). Twelve SNPs were significantly associated with lint percentage; these SNP loci were positioned on chromosomes A_t_3 (3), A_t_4 (4), A_t_10 (1), A_t_11 (2), and D_t_3 (2) (Figure 2A). Most importantly, five of the twelve SNPs significantly associated with the trait were detected with the lowest *P*-value (−log_10_(*P*) > 6.21); these five loci were positioned on chromosomes A_t_3 and A_t_4 of upland cotton and explained 9.42–12.88% of the total phenotypic variance (Figure 2A, Table 1). Interestingly, two SNP loci (rsA_t_4:15572813 and rsA_t_4:15573052) on chromosome A_t_4 showed significant marker-trait associations with the highest −log_10_(*P*) values, which were detected in BLUP and each of the four environments simultaneously (Figure 2A, Supplementary Figure S1, and Table 1). Additionally, three SNP loci (rsA_t_3:43538238, rsA_t_3:43631774, and rsA_t_3:43631819) on chromosome A_t_3 were associated with the target trait in BLUP and three of the four environments at the same time (Figure 2A, Supplementary Figure S1, and Table 1). These results indicated that there were two major-effect QTLs for lint percentage on chromosomes A_t_3 and A_t_4.

**Table 1:** **SNPs significantly associated with lint percentage by the MLM**.

**ID**	**SNPs^a^**	**Chr**.	**Position (Mbp)**	**Allele^b^**	**Environments**									
					**BLUP**		**AY-14**		**AY-15**		**SHZ-14**		**SHZ-15**	
					**−log_10_(*P*)**	***R*^2^(%)**	**−log_10_(*P*)**	***R*^2^(%)**	**−log_10_(*P*)**	***R*^2^(%)**	**−log_10_(*P*)**	***R*^2^(%)**	**−log_10_(*P*)**	***R*^2^(%)**
1	rsA_t_3:43538238	A_t_3	43.54	G/T	6.56	9.42	5.97	8.01			7.06	9.62	6.36	8.55
2	rsA_t_3:43631774	A_t_3	43.63	C/T	7.43	10.80	7.29	10.00			7.66	10.52	6.70	9.15
3	rsA_t_3:43631819	A_t_3	43.63	A/G	7.42	10.79	6.97	9.53			7.55	10.37	6.61	9.03
4	rsA_t_4:15572813	A_t_4	15.57	A/G	8.15	11.94	7.24	10.14	6.52	8.97	6.88	9.32	6.38	8.61
5	rsA_t_4:15573052	A_t_4	15.57	A/G	8.72	12.88	8.13	11.39	6.59	9.11	7.65	10.41	6.70	9.13

a *Only the SNPs associated significantly with the BLUPs for lint percentage across four environments (−log_10_P > 6.21) are shown*.

b *Major and minor alleles; the favorable alleles are underlined*.

### Identification of favorable haplotypes (FHs) of two major-effect QTLs for lint percentage

**Figure 3:**
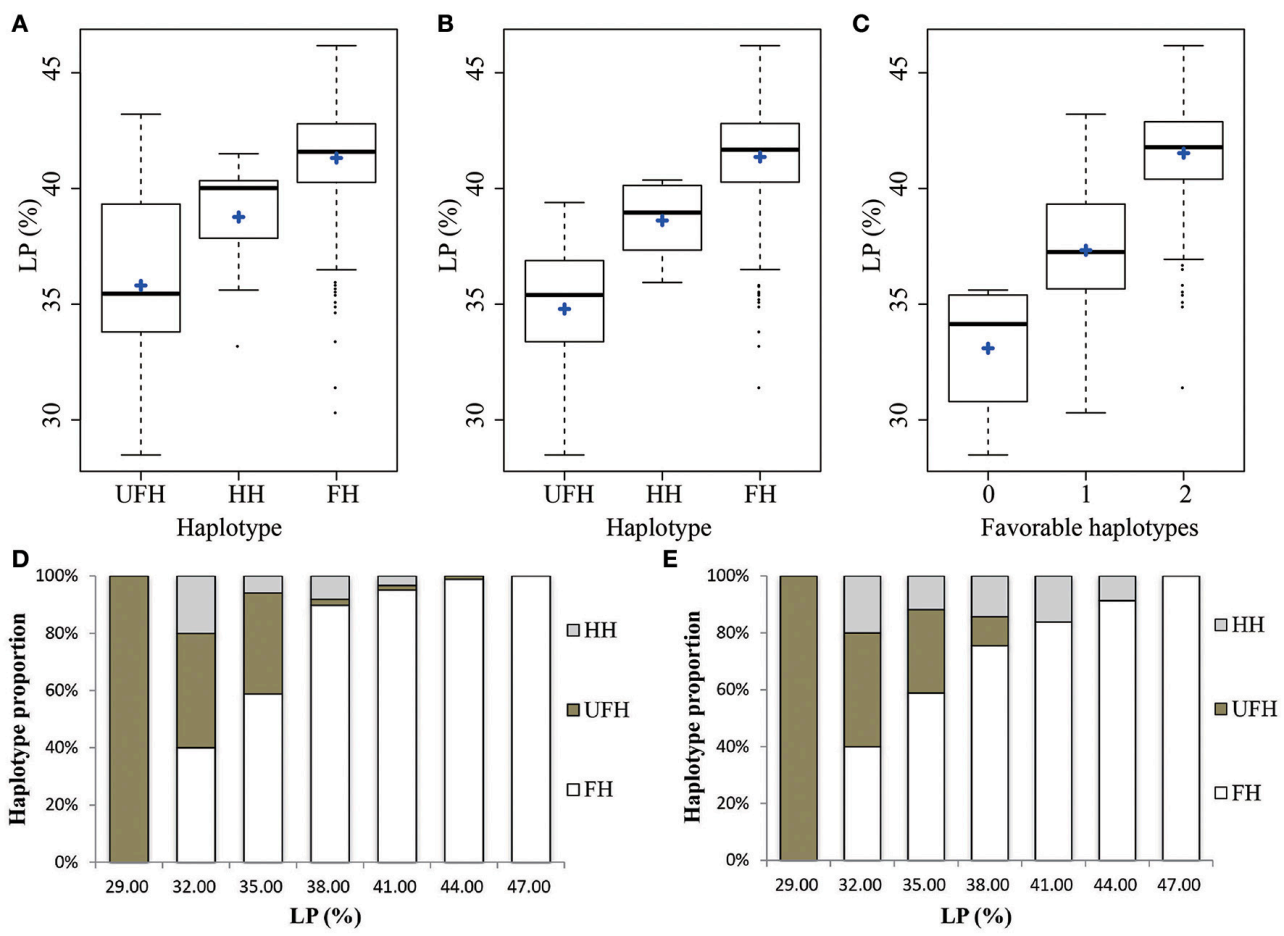
**Phenotypic distribution of the haplotypes of two major-effect QTLs for lint percentage. (A,B)** Box plots for the phenotypic values of the major-effect QTLs on chromosomes A_t_3 and A_t_4, respectively. **(C)** Box plot for lint percentage plotted as the favorable haplotype number. **(D,E)** Charts of several types of haplotypes accounting for proportion. FHs, favorable haplotypes; UFHs, unfavorable haplotypes; HHs, heterozygous haplotypes; LP, lint percentage. The middle line is the median, the blue plus sign indicates the mean, the box represents the range from the 25th to 75th percentiles of the total data, the whiskers represent the inter-quartile range, and the outer dots are outliers.

To identify favorable QTL alleles for lint percentage, the five significantly associated SNP loci (−log_10_(*P*) > 6.21) in two major-effect QTLs were selected, which exhibited the minimum *P*-value and could explain the maximum phenotypic variation. Three SNP loci from chromosome A_t_3 (rsA_*t*_3:43538238, rsA_t_3:43631774, and rsA_t_3:43631819) were associated with lint percentage via GWAS; these SNP alleles were G/T, C/T, and A/G, respectively. Three types of haplotypes (GG-TT-GG, TT-CC-AA, and GT-CT-AG) were found because of a close linkage relationship among the three SNP loci. The GG-TT-GG haplotype included 308 lines and was considered the favorable haplotype (FH) because the average lint percentage (41.32%) of the haplotype was significantly higher than the mean value (35.81%) of the other corresponding haplotype (TT-CC-AA, the unfavorable haplotype, UFH) and included 26 lines. The remaining 21 lines were heterozygous haplotypes (HH, GT-CT-AG), with a mean lint percentage of 38.77% (Figure 3A). Furthermore, the FH accounted for a very large proportion of the accessions with higher lint percentage than that with lower lint percentage. For example, there was no FH in the accessions with a low lint percentage (< 30.50%), and there was no UFH in the lines with a high lint percentage (>45.50%), (Figure 3D). The other two SNP loci (rsA_t_4:15572813 and rsA_t_4:15573052) associated with the lint percentage were closely linked and were located on chromosome A_t_4, separated by 238 bp. Similarly, the lint percentage of accessions with the FH (AA-AA, 41.36%), was higher on average than that of accessions with the UFH (GG-GG, 34.79%; Figure 3B). The FH accounted for a large proportion of the accessions with a higher lint percentage, and the UFH was not found among the upland cotton lines with lint percentage values >39.50% (Figure 3E). These results further confirmed that there were two major QTLs controlling lint percentage in the adjacent regions of the five associated SNP loci.

To further understand the cumulative effect of FHs on lint percentage, the 355 upland cotton lines were grouped into three groups according to the number of FHs. The cotton lint percentage among the three groups obviously increased with an increasing number of FHs (Figure 3C). For example, accessions with two FHs showed a 41.53% mean lint percentage, which was higher than accessions with one FH (37.33%) and those without a FH (33.09%). These results suggested that the genetic control of lint percentage exhibits a largely additive effect in upland cotton.

### Prediction of candidate genes

Two upland cotton (TM-1) reference genome sequences are currently available in a public database (http://www.cottongen.org). To identify the corresponding chromosomal locations of the SNP loci associated with the target trait between two upland cotton reference genomes, 250 bp of the sequence on either side of each associated SNP were extracted from one upland cotton reference genome (Li et al., 2015), and 501 bp of the sequences was aligned with the other upland cotton reference genome (Zhang et al., 2015). There was a clear correspondence between the chromosomal positions of the SNP loci associated with lint percentage between these two upland cotton reference genomes. For example, positions 15572813 and 15573052 on chromosome A_t_4 corresponded to 52098956 and 52098717 on chromosome A08, respectively, while positions 43538238, 43631774 and 43631819 on chromosome A_t_3 corresponded to 74801531, 74713290 and 74713245 on chromosome A02, respectively (Table 2).

**Table 2:** **Corresponding chromosomal positions for the SNP loci associated with lint percentage between two upland cotton reference genomes**.

**SNP markers**	**Chromosome^a^**	**Position^a^**	**Chromosome^b^**	**Position^b^**	**Linkage group**
rsAt4:15572813	At4	15572813	A08	52098956	c4
reAt4:15573052	At4	15573052	A08	52098717	c4
rsAt3:43538238	At3	43538238	A02	74801531	c3
rsAt3:43631774	At3	43631774	A02	74713290	c3
rsAt3:43631819	At3	43631819	A02	74713245	c3
rsAt11:9054901	At11	9054901	A10	85557206	c10
rsDt3:18809554	Dt3	18809554	D03	39463548	c17
rsDt3:18792729	Dt3	18792729	D03	39445829	c17
rsAt4:57048135	At4	57048135	scaffold2053_A08	54582	c4
rsAt4:30206539	At4	30206539	scaffold2020_A08	59124	c4
rsAt11:55272721	At11	55272721			
rsAt10:20706257	At10	20706257	A06	85019634	c6

a *The upland cotton reference genome according to Li et al. (2015)*.

b *The upland cotton reference genome according to Zhang et al. (2015)*.

**Figure 4:**
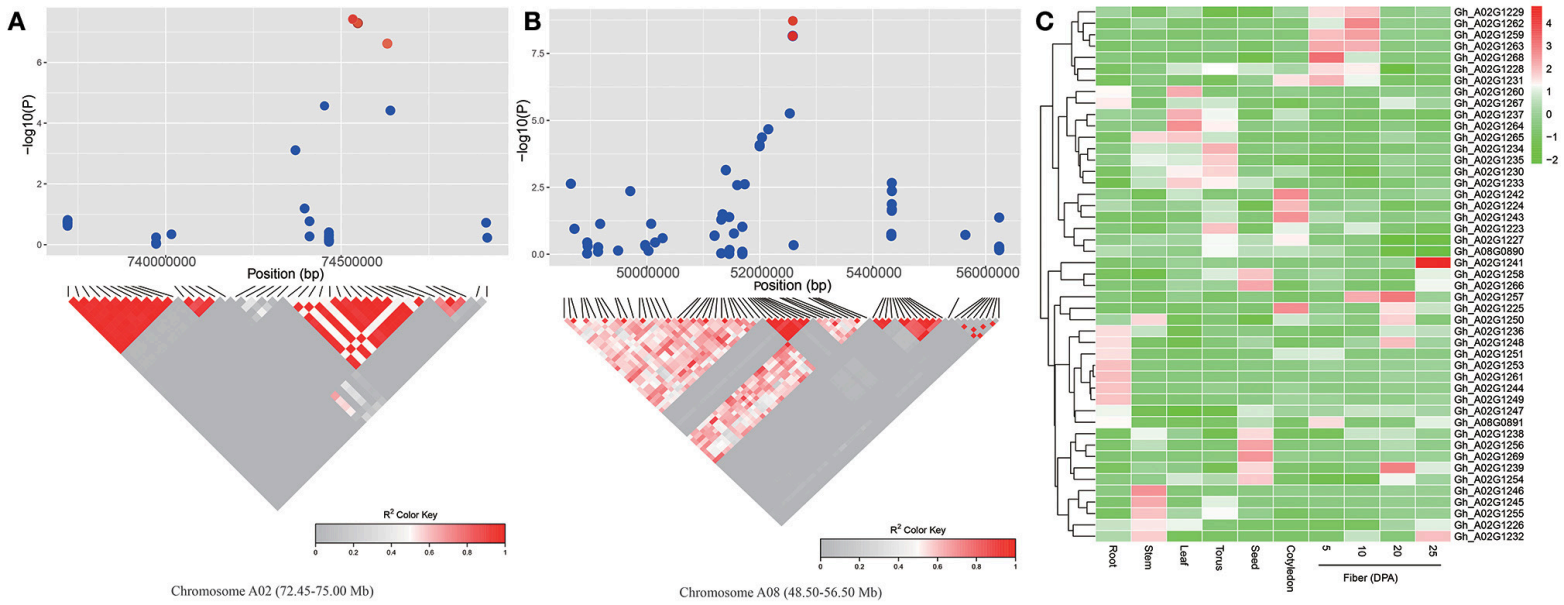
**Prediction of the candidate genes for lint percentage. (A)** The peak region (72.45–75.00 Mb) on chromosome A02. **(B)** The peak region (48.50–56.50 Mb) on chromosome A08. **(A,B)** The pair-wise LD between the SNP markers is indicated as D' values, where dark red indicates a value of 1 and gray indicates 0. The dotted squares in **(A,B)** indicate the LD blocks that contain significant SNPs (red dot). **(C)** Heat map of the putative candidate gene expression patterns in 10 upland cotton (TM-1) tissues. Red indicates high expression, and green indicates low expression.

To look for putative candidate genes in the neighboring regions of the SNP loci associated with lint percentage, we further determined LD blocks harboring five significant SNPs (−log_10_(*P*) > 6.21). Two LD blocks were found on chromosomes A02 (73.25–74.75 Mb) and A08 (51.95–52.12 Mb) (Figures 4A,B). Based on the results of the genes annotated in the CottonGen database (http://www.cottongen.org), a total of 45 and 2 putative candidate genes were contained in two LD blocks on chromosomes A02 and A08, respectively. Furthermore, the expression levels of 47 putative candidate genes were analyzed using RNA-seq data from ten upland cotton (TM-1) tissues (Zhang et al., 2015). Six genes (*Gh_A02G1229, Gh_A02G1262, Gh_A02G1259, Gh_A02G1263, Gh_A02G1268*, and *Gh_A02G1228*) presented higher expression levels in the fiber-5 DPA and fiber-10 DPA than in the other eight tissues. Specifically, only one gene (*Gh_A02G1268*) was predominantly expressed in fiber-5 DPA, and its expression levels in fiber-5 DPA were 300-fold higher than in the seeds in TM-1 (Figure 4C), suggesting its potential role in increasing the lint percentage and improving fiber quality in upland cotton. *Gh_A02G1268*, a member of the myo-inositol-1-phosphate synthase (*MIPS*) gene family, was located 95.81 kb away from the peak SNP (A02:74713245). Its closest *Arabidopsis* homolog (*At2g22240*) is known to regulate embryogenesis and seed development (Mitsuhashi et al., 2008; Luo et al., 2011).

### Comparison of the GWAS with QTLs identified in previous studies

**Figure 5:**
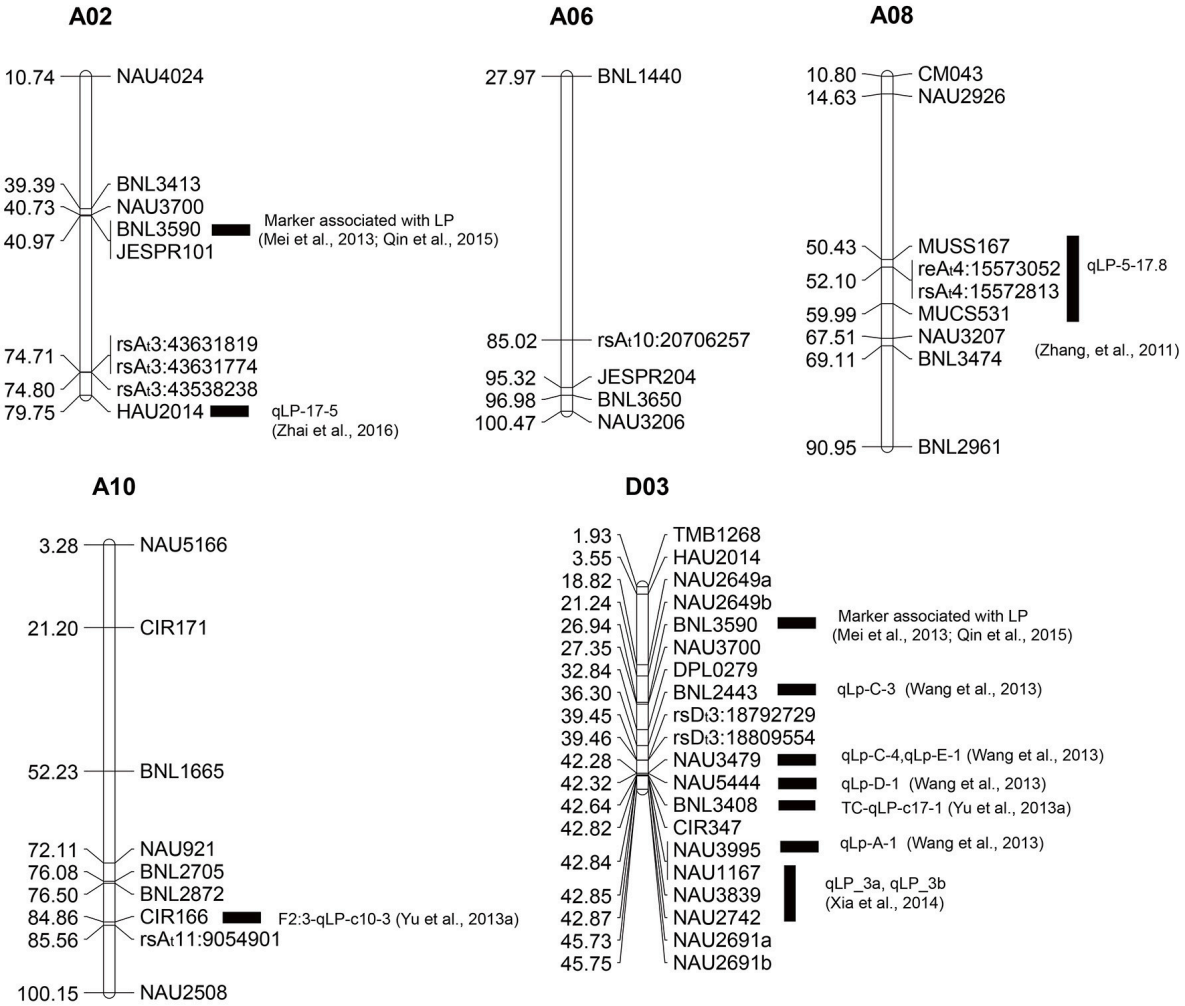
**Physical map of molecular markers identified on five chromosomes (A02, A06 A08, A10, and D03) based on our GWAS and QTL and association mapping from previous studies**. The unit of physical distance for the chromosomes is Mb; black rectangles represent SSR markers or QTLs from previous studies in the vicinity of the associated SNP loci identified through our GWAS.

To further confirm these SNP loci associated with the lint percentage, our new GWAS was compared with previous linkage and association studies. Regarding previous QTL mapping studies, 281 SSR markers (Supplementary Tables S4, S5) containing QTLs for lint percentage were selected from 23 reports of QTL mapping (Supplementary Table S6), and 183 primer sequences corresponding to these markers (Supplementary Table S4) were obtained from the CottonGen Database (http://www.cottongen.org). The physical locations of these SSR primer sequences were mapped to the reference genome sequence (Zhang et al., 2015) via electronic PCR (e-PCR), among which 6, 4, 7, 8, and 18 SSR primers were mapped to chromosomes A02, A06 A08, A10, and D03, respectively (Figure 5). Three SNP loci from chromosome A02 (A_t_3) (rsA_t_3:43538238, rsA_t_3:43631774, and rsA_t_3:43631819) were mapped to an adjacent region of HAU2014; two SNP loci (rsA_t_4:15572813 and rsA_t_4:15573052) from chromosome A08 (A_t_4) were positioned between MUSS167 and MUCS531; rsA_t_11:9054901 near CIR166 was located on chromosome A10 (A_t_11); and two SNP loci (rsD_t_3:18792729 and rsD_t_3:18809554) from chromosome D03 (D_t_3) were mapped in the vicinity of many QTL-SSR markers, such as BNL2443, NAU3479 and NAU5444.

## Discussion

The power to resolve associated loci for a target trait via GWAS depends on the marker density, the experimental population size and statistical methods (Liu et al., 2016). Association analyses based on SSR markers have been widely reported in upland cotton (Abdurakhmonov et al., 2008; Mei et al., 2013; Qin et al., 2015; Nie et al., 2016). Sequencing technologies, such as GBS, RAD-seq, and SLAF-seq, are now available for the development of abundant SNPs in upland cotton (Gore et al., 2014; Chen et al., 2015; Wang H. et al., 2015; Wang Y. et al., 2015; Zhang et al., 2016). Therefore, development of SNP markers using GBS, RAD-seq and SLAF-seq can currently meet the needs of GWAS in cotton. The decay of LD over physical distances in a population determines the density of the marker coverage needed to perform a GWAS (Yu and Buckler, 2006). The faster LD decays, the more markers are likely needed for GWAS for complex traits. In previous studies, the average distance of LD decay was 3–7 cM, and 100–400 SSR markers were used for statistical analysis in upland cotton (Abdurakhmonov et al., 2008; Fang et al., 2013; Mei et al., 2013; Cai et al., 2014; Qin et al., 2015; Nie et al., 2016). In our study, a considerable number of markers (81,675 SNPs) were identified by SLAF-seq, and a mean LD decay distance of 100 kb was estimated in the population. Considering the LD status in the panel of upland cotton lines, the mean marker density (1 SNP per 24.85 kb) in this study was sufficient to perform a GWAS. Population size is another determinant affecting the power of GWAS. It has been shown that increasing the population size has a greater influence on the power of QTL detection than increasing the SNP marker density (Long and Langley, 1999). Indeed, increasing the number of population individuals would likely identify more smaller-effect QTLs (Zhu et al., 2008). Population sizes ranging from 100 to 500 have been used for association analysis in upland cotton in previous studies (Abdurakhmonov et al., 2008; Mei et al., 2013; Qin et al., 2015; Nie et al., 2016). In our study, although a population with 355 diverse accessions was not sufficiently large, the phenotypic variations in the lint percentage were very large, ranging from 26.58 to 48.13%. At present, MLM, which simultaneously estimates the population structure and unequal relatedness among individuals, is a widely used statistical model in plant association mapping and allows a large reduction in spurious associations (Yu et al., 2006). It has been recently suggested that PCA is a fast and effective way to infer population structure (Yu et al., 2006). Using PCA to estimate population structure was better than using the Q model for controlling false positives (Zhao et al., 2007; Xu et al., 2016). In this study, to further control for false positives, MLM was selected for association mapping by incorporating PCs and kinship data, and some SNP loci that significantly associated with lint percentage throughout the upland cotton genome could be identified at one time.

The phenotypes of complex traits often result from the combined actions of multiple genes and environmental factors, all of which can easily lead to lost heritability (Mackay et al., 2009). Therefore, only those traits with high heritability can be stably detected. The stably associated markers with target traits should be useful for cotton breeding with broad adaptability to different environments (Mei et al., 2013). In our study, we found that all five SNPs significantly associated with lint percentage were detected in BULP and three or more environments, which was consistent with the phenotypic statistical analysis that lint percentage demonstrated a particularly high broad-sense heritability (69.72%). Linkage mapping was the uppermost method for QTL identification. Previous linkage mapping identified at least 25 distinct QTLs for lint percentage distributed in 14 linkage groups in tetraploid cotton (Said et al., 2013). Seven hotspots (c3-mQTL-LP-Gh-1: 0-20 cM, c3-mQTL-LP-Gh-2: 25-45 cM, c7-mQTL-LP-Gh-3: 0-20 cM, c11-mQTL-LP-Gh-4: 0-20 cM, c13-mQTL-LP-Gh-5: 39-60 cM, c16-mQTL-LP-Gh-6: 0-23 cM and c24-mQTL-LP-Gh-7: 0-20 cM) for lint percentage QTLs were identified in intraspecific *G. hirsutum* populations (Said et al., 2015). Association mapping can serve as a complement for linkage mapping and may identify more loci or QTLs because linkage mapping detects only QTLs that exist as polymorphisms in the parents. Using association mapping, a number of SSR loci associated with lint percentage were detected. For example, 31 SSR markers distributed on 20 chromosomes were shown to be significantly associated with lint percentage (Mei et al., 2013; Qin et al., 2015). In this study, 12 associated loci were detected based on GWAS (Table 2), and these SNP loci were positioned on chromosomes A_t_3 (A02), A_t_4 (A08), A_t_10 (A06), A_t_11 (A10), and D_t_3 (D03). We found that 10/12 (83.33%) SNP loci associated with lint percentage were distributed on the A subgenome. Interestingly, two SNP loci (rsA_t_4:15572813 and rsA_t_4:15573052) on chromosome A_t_4 (A08) with the highest −log_10_(*P*) values were distributed within a QTL (qLP-5-17.8) identified in a previous study (Zhang et al., 2011), and two SNP loci (rsD_t_3:18792729 and rsD_t_3:18809554) from chromosome D03 (D_t_3) were mapped in the vicinity of five QTLs (qLp-A-1 qLp-C-3 qLp-C-4 qLp-D-1 and qLp-E-1) located on chromosome A3 (Wang et al., 2013). These findings validate the GWAS results and increase confidence in the identity of some SNP loci. In addition, we found the SSR marker BNL3590 mapped to A02:40,971,023-40,971,204 bp and D03: 26,940,735- 26,940,924 bp in the reference genome sequence (Zhang et al., 2015) by e-PCR. Although, the SSR marker BNL3590 was significantly associated with the lint percentage in previous studies (Mei et al., 2013; Qin et al., 2015), it was not in the associated genome region (A02: 74801531-74713245) identified in our study. We also found that accumulating FHs might be an effective way to increase the lint percentage during the selection of high lint percentage lines. Genotypic classification revealed that the cotton lint percentage can be increased by pyramiding the FHs. This result strongly suggests that lint percentage is largely controlled by quantitative genes with mainly additive effects.

Cotton fiber development is a complex process that involves fiber initiation, elongation, secondary cell wall synthesis, and maturation (Kim and Triplett, 2001). Long lint fibers are initiated before or on the day of anthesis (Meinert and Delmer, 1977), and most of the genes showing significant differences are found during earlier stages of fiber development (Al-Ghazi et al., 2009). Previous research results suggested that different final fiber properties may be established at earlier stages of fiber development. Two determinant factors for cotton lint percentage are seed and fiber. Similarly, we hypothesized that high expression of genes controlling the lint percentage would be observed at earlier stages in fiber development. In our study, six genes (*Gh_A02G1229, Gh_A02G1262, Gh_A02G1259, Gh_A02G1263, Gh_A02G1268*, and *Gh_A02G1228*) in two LD blocks containing five significant SNPs showed higher expression levels at earlier stages during fiber development. The previous studies showed that plant *MIPS* genes were highly expressed in developing seeds, suggesting that myo-inositol plays an important role in seed/embryo development (Johnson and Wang, 1996; Hegeman et al., 2001). In this study, *Gh_A02G1268* (*MIPS*) was very highly expressed in fiber-5 DPA, while the gene was poorly expressed in TM-1 seeds. These results implied that *Gh_A02G1268* may determine lint percentage by regulating seed and fiber development and indicated that *Gh_A02G1268* was a candidate gene for QTLs that control the lint percentage trait.

## Conclusion

In this study, 12 SNP loci associated with lint percentage were identified via GWAS, in which five SNP loci that significantly associated with the trait were detected with the lowest *P*-value (−log_10_(*P*) > 6.21). These five loci were located on chromosomes A_t_3 (A02) and A_t_4 (A08) of upland cotton and explained 9.42–12.88% of the total phenotypic variance. The favorable haplotypes (FHs) for two major-effect QTLs for lint percentage were identified and 47 putative candidate genes were annotated in two LD blocks harboring five significant SNPs. By analyzing RNA-seq data, we found that *Gh_A02G1268* was very highly expressed during an early fiber development stage and poorly expressed in the TM-1 seed. These results implied that *Gh_A02G1268* may determine lint percentage by regulating seed and fiber development. The favorable QTL alleles and candidate genes for lint percentage identified in this study show high potential for the improvement of lint yield in future cotton breeding programs.

## Author contributions

SY, CP designed and supervised the research. JS, CP, LL, and HLW analyzed the data. JS, LL, SF, CZ, SZ, and GM conducted the field trials to evaluate the traits. LL, CSW, and LG performed the data analysis. JS, CXW wrote the manuscript. All of the authors read and approved the manuscript.

### Conflict of interest statement

The authors declare that the research was conducted in the absence of any commercial or financial relationships that could be construed as a potential conflict of interest.

## Supplementary material

Supplementary Figure S1 **Manhattan plots of the genome-wide association studies (GWASs) for each environment for lint percentage. (A–D)** represent the AY-14, AY-15, SHZ-14, and SHZ-15 environments, respectively. Each dot represents a SNP. The horizontal dotted red and blue lines indicate the Bonferroni-corrected significance thresholds at −log_10_(P) = 5.21 and −log_10_(P) = 6.21, respectively.

Supplementary Table S1 **Information on the 355 upland cotton accessions**.

Supplementary Table S2 **Descriptive statistics for the lint percentage in the four different environments**.

Supplementary Table S3 **Correlations between the different environments based on the 355 upland cotton accessions**.

Supplementary Table S4 **The 183 SSR primers whose sequences were obtained from public databases**.

Supplementary Table S5 **The 98 SSR primers whose sequences were not obtained from public databases**.

Supplementary Table S6 **The QTLs of FL and FS from 23 reports of QTL mapping**.
