# Supplementary figures and images for: Detection of Favorable QTL Alleles and Candidate Genes for Lint Percentage by GWAS in Chinese Upland Cotton
Source: Front Plant Sci. 2016 Oct 21;7:1576. doi: 10.3389/fpls.2016.01576 (PMC5073211; doi:10.3389/fpls.2016.01576)

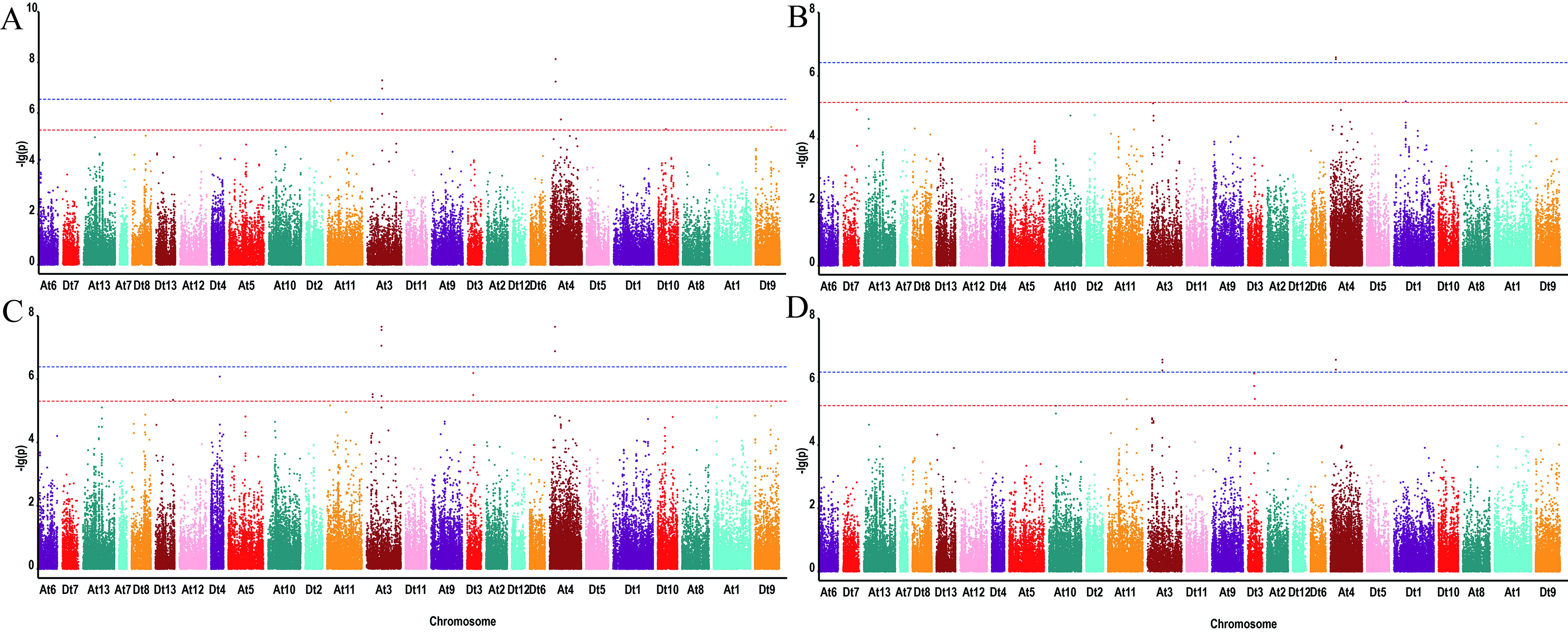

Supplement: Supplementary Figure S1 — Manhattan plots of the genome-wide association studies (GWASs) for each environment for lint percentage. (A–D) represent the AY-14, AY-15, SHZ-14, and SHZ-15 environments, respectively. Each dot represents a SNP. The horizontal dotted red and blue lines indicate the Bonferroni-corrected significance thresholds at −log10(P) = 5.21 and −log10(P) = 6.21, respectively. [file Image1.JPEG]
