# Supplementary material for: Detection of Favorable QTL Alleles and Candidate Genes for Lint Percentage by GWAS in Chinese Upland Cotton
Source: Front Plant Sci. 2016 Oct 21;7:1576. doi: 10.3389/fpls.2016.01576 (PMC5073211; doi:10.3389/fpls.2016.01576)
Supplement: Supplementary Table S1 — Information on the 355 upland cotton accessions. [file Table1.DOCX]

Supplementary Table S1. Information on 355 upland cotton germplasms.

| Serial  number | Variety  names | Geographic  origin | Serial  number | Variety  names | Geographic  origin | Serial  number | Variety  names | Geographic  origin |
| --- | --- | --- | --- | --- | --- | --- | --- | --- |
| 1 | zhong02191 | HY | 51 | liaomian7 | LN | 101 | zhongmiansuo64 | HY |
| 2 | zhong040418 | HY | 52 | liaomian9 | LN | 102 | zhongmiansuo74 | HY |
| 3 | zhong040618 | HY | 53 | liaoyangduanjie | LN | 103 | 776 | HY |
| 4 | zhong040712 | HY | 54 | lu154 | HY | 104 | 20398 | HY |
| 5 | zhong051811 | HY | 55 | lu890 | HY | 105 | 61930 | HY |
| 6 | zhong051822 | HY | 56 | lumina2153 | HY | 106 | 61995 | HY |
| 7 | zhong061832 | HY | 57 | nongken5 | NW | 107 | 102909 | HY |
| 8 | zhong071239 | HY | 58 | shan70 | HY | 108 | 103026 | HY |
| 9 | PB12-1-10 | HY | 59 | shizao1 | HY | 109 | 103028 | HY |
| 10 | PB12-1-7 | HY | 60 | shizao2 | HY | 110 | 103030 | HY |
| 11 | PB12-1-8 | HY | 61 | shizao3 | HY | 111 | 103032 | HY |
| 12 | zhong1476 | HY | 62 | xia13-7 | HY | 112 | 103075 | HY |
| 13 | zhong151222 | HY | 63 | xia25 | HY | 113 | 103164 | HY |
| 14 | zhong152201 | HY | 64 | xiazao1 | HY | 114 | 103245 | HY |
| 15 | SQ152201 | HY | 65 | xiazao2 | HY | 115 | 103297 | HY |
| 16 | zhong152214 | HY | 66 | xiazao3 | HY | 116 | 109056 | HY |
| 17 | SQ152224 | HY | 67 | xinluzao11 | NW | 117 | SPPB12-1-7 | HY |
| 18 | 2011SS | HY | 68 | xinluzao36 | NW | 118 | SPPB12-1-9 | HY |
| 19 | QS2012-3 | HY | 69 | xinluzao3 | NW | 119 | SPPB12-2-7 | HY |
| 20 | QS2012-4 | HY | 70 | xinluzao42 | NW | 120 | 298 | HY |
| 21 | 29-41 | HY | 71 | xinluzao45 | NW | 121 | 602186 | HY |
| 22 | 29-42 | HY | 72 | xinluzao4 | NW | 122 | CG3020-1 | USA |
| 23 | 6426 | HY | 73 | xinluzao6 | NW | 123 | CG3020-3 | USA |
| 24 | K640 | HZY | 74 | xinluzao8 | NW | 124 | FM1735 | USA |
| 25 | N82 | HZY | 75 | xinluzao9 | NW | 125 | G2005 | HY |
| 26 | P21-6-7 | HZY | 76 | xinxiang368 | HY | 126 | GK44 | HY |
| 27 | SF06 | HY | 77 | yu1335 | HY | 127 | H109 | HY |
| 28 | SGK16 | HZY | 78 | yumian12 | HY | 128 | H559 | HY |
| 29 | V321-20-14 | HZY | 79 | yuzao8E13 | HY | 129 | LIH33 | HY |
| 30 | baimian17 | HY | 80 | yuzao910 | HY | 130 | Phy-7 | USA |
| 31 | chaoyangmian1 | LN | 81 | yunzaoN177 | HY | 131 | STS458 | USA |
| 32 | Deltapine20 | USA | 82 | yunzaoN95 | HY | 132 | TM-1 | USA |
| 33 | Delfos97-047 | USA | 83 | zhong416 | HY | 133 | aoshimian4406 | HY |
| 34 | guannong1 | LN | 84 | zhong425-5 | HY | 134 | baimian985 | HY |
| 35 | han2490 | HY | 85 | zhong716 | HY | 135 | cang198 | HY |
| 36 | han656 | HY | 86 | zhong751213 | HY | 136 | chunbeibao | HY |
| 37 | han559 | HY | 87 | zhongchuang88 | HY | 137 | chunnanbao | HY |
| 38 | han667 | HY | 88 | zhongmiansuo10 | HY | 138 | Deltapine14 | USA |
| 39 | han686 | HY | 89 | zhongmiansuo14 | HY | 139 | Deltapine15 | USA |
| 40 | han9609 | HY | 90 | zhongmiansuo16 | HY | 140 | fanmian3 | HY |
| 41 | heishanmian1 | LN | 91 | zhongmiansuo20 | HY | 141 | ganzao109 | HZY |
| 42 | jinmian3 | LN | 92 | zhongmiansuo24 | HY | 142 | guoxinmian11 | HY |
| 43 | jinmian10 | HY | 93 | zhongmiansuo27 | HY | 143 | han7860 | HY |
| 44 | jinmian21 | HY | 94 | zhongmiansuo30 | HY | 144 | ji4025 | HY |
| 45 | jinmian23 | HY | 95 | zhongmiansuo36 | HY | 145 | jinmian26 | HY |
| 46 | jinmian5 | HY | 96 | han256 | HY | 146 | BM03 | HY |
| 47 | liaomian10 | LN | 97 | zhongmiansuo37 | HY | 147 | kelin098 | HY |
| 48 | liaomian17 | LN | 98 | zhongmiansuo42 | HY | 148 | liaomian23 | LN |
| 49 | liaomian5 | LN | 99 | zhongmiansuo50 | HY | 149 | liaomian27 | LN |
| 50 | liaomian6 | LN | 100 | zhongmiansuo58 | HY | 150 | liaomian28 | LN |

| 151 | lu05R59 | HY | 201 | chuan01 | HZY | 251 | xinluzhong6 | NW |
| --- | --- | --- | --- | --- | --- | --- | --- | --- |
| 152 | lu7619 | HY | 202 | xinluzao2 | NW | 252 | xinluzhong7 | NW |
| 153 | lumianyan17 | HY | 203 | xinluzao10 | NW | 253 | xinluzhong8 | NW |
| 154 | lumianyan21 | HY | 204 | xinluzao12 | NW | 254 | xinluzhong9 | NW |
| 155 | lumianyan28 | HY | 205 | xinluzao13 | NW | 255 | xinluzhong10 | NW |
| 156 | lumianyan36 | HY | 206 | xinluzao15 | NW | 256 | xinluzhong12 | NW |
| 157 | lumianyan38 | HY | 207 | xinluzao16 | NW | 257 | xinluzhong13 | NW |
| 158 | miaobao21 | HY | 208 | xinluzao17 | NW | 258 | xinluzhong14 | NW |
| 159 | renhe39 | HY | 209 | xinluzao18 | NW | 259 | xinluzhong15 | NW |
| 160 | rihuimian6 | HZY | 210 | xinluzao19 | NW | 260 | xinluzhong16 | NW |
| 161 | shannongSF01 | HY | 211 | xinluzao20 | NW | 261 | xinluzhong17 | NW |
| 162 | shan79 | HY | 212 | xinluzao21 | NW | 262 | xinluzhong19 | NW |
| 163 | Stoneville2B | USA | 213 | xinluzao22 | NW | 263 | xinluzhong20 | NW |
| 164 | xinmian33B | USA | 214 | xinluzao23 | NW | 264 | xinluzhong21 | NW |
| 165 | xinzhimian5 | HY | 215 | xinluzao24 | NW | 265 | xinluzhong22 | NW |
| 166 | yinhuashu | HY | 216 | yumian5 | HY | 266 | xinluzhong26 | NW |
| 167 | you009 | HY | 217 | yumian18 | HY | 267 | xinluzhong27 | NW |
| 168 | zhong109 | HY | 218 | yumian21 | HY | 268 | xinluzhong28 | NW |
| 169 | zhongmiansuo17 | HY | 219 | yun1729 | HY | 269 | xinluzhong30 | NW |
| 170 | zhongmiansuo19 | HY | 220 | zhemian11 | HZY | 270 | xinluzhong32 | NW |
| 171 | zhongmiansuo43 | HY | 221 | Tkuo | NW | 271 | xinluzhong34 | NW |
| 172 | zhongmiansuo60 | HY | 222 | bo425 | NW | 272 | xinluzhong35 | NW |
| 173 | zhong662 | HY | 223 | xinluzhong60 | NW | 273 | xinluzhong40 | NW |
| 174 | zhong679 | HY | 224 | kenN27-3 | NW | 274 | xinluzhong41 | NW |
| 175 | zhongmiansuo69 | HY | 225 | B-3 | NW | 275 | xinluzhong45 | NW |
| 176 | zhong800319 | HY | 226 | xinluzao25 | NW | 276 | xinluzhong46 | NW |
| 177 | zhong915 | HY | 227 | xinluzao26 | NW | 277 | xinluzhong47 | NW |
| 178 | zhongmiansuo12 | HY | 228 | xinluzao27 | NW | 278 | KZGL | USA |
| 179 | zhongmiansuo35 | HY | 229 | xinluzao28 | NW | 279 | jiangyin1 | HZY |
| 180 | zhongmiansuo41 | HY | 230 | xinluzao29 | NW | 280 | huihe36 | NW |
| 181 | zhongmiansuo45 | HY | 231 | xinluzao30 | NW | 281 | xinluzao60 | NW |
| 182 | zhongmiansuo49 | HY | 232 | xinluzao32 | NW | 282 | guoxinmian9 | HY |
| 183 | zhongmiansuo7 | HY | 233 | xinluzao33 | NW | 283 | junmian1 | NW |
| 184 | zhongzhimian8 | HY | 234 | xinluzao34 | NW | 284 | huiyuan717 | NW |
| 185 | zhongzhimianGD89 | HY | 235 | xinluzao35 | NW | 285 | yunzao219 | HY |
| 186 | shan920346 | HY | 236 | xinluzao37 | NW | 286 | yunzao33-356 | HY |
| 187 | xianⅢ9704 | NW | 237 | xinluzao38 | NW | 287 | jinmian2 | LN |
| 188 | USA-1 | USA | 238 | xinluzao39 | NW | 288 | chaoyangmian2 | LN |
| 189 | JEJS | Others | 239 | xinluzao40 | NW | 289 | dunmian77-116 | NW |
| 190 | bazhou5409 | NW | 240 | xinluzao41 | NW | 290 | ganmian4 | NW |
| 191 | yumian1 | HZY | 241 | xinluzao46 | NW | 291 | guannongzaoC-50 | LN |
| 192 | huazhong910102 | HZY | 242 | xinluzao47 | NW | 292 | gunagnongchangzao14 | LN |
| 193 | yiselie | Others | 243 | xinluzao48 | NW | 293 | yanzao1 | HZY |
| 194 | ken6614 | NW | 244 | xinluzao49 | NW | 294 | yanzao2 | HZY |
| 195 | ken0074 | NW | 245 | xinluzao50 | NW | 295 | liaojinmian6 | LN |
| 196 | chuan239-1 | HZY | 246 | xinluzao51 | NW | 296 | jinken69-2 | NW |
| 197 | bamian3号 | NW | 247 | xinluzhong1 | NW | 297 | jinken148-39 | NW |
| 198 | chuan338 | HZY | 248 | xinluzhong3 | NW | 298 | zhuangjiahan102 | NW |
| 199 | chaun267 | HZY | 249 | xinluzhong4 | NW | 299 | yinmian4 | HY |
| 200 | chuan65 | HZY | 250 | xinluzhong5 | NW | 300 | ejing1 | HZY |

| 301 | simian3 | HZY | 326 | xiangmian13 | HZY | 351 | xuzhou219 | HZY |
| --- | --- | --- | --- | --- | --- | --- | --- | --- |
| 302 | Stoneville4B | USA | 327 | yapengmian | HZY | 352 | yinmian8 | HY |
| 303 | jimian10 | HY | 328 | jijiaodezimian | USA | 353 | yumian1 | HY |
| 304 | jimian11 | HY | 329 | 611bo | Others | 354 | yumian2 | HY |
| 305 | jimian12 | HY | 330 | annong121 | HZY | 355 | xinluzao53 | NW |
| 306 | emian16 | HZY | 331 | daizimian16 | USA |  |  |  |
| 307 | emian17 | HZY | 332 | dezimian531 | USA |  |  |  |
| 308 | sumian4 | HZY | 333 | dunmian1 | NW |  |  |  |
| 309 | ekangmian2 | HZY | 334 | dunmian2 | NW |  |  |  |
| 310 | ekangmian3 | HZY | 335 | ganmian2 | HZY |  |  |  |
| 311 | ekangmian6 | HZY | 336 | ganmian3 | HZY |  |  |  |
| 312 | edaimian | HZY | 337 | ganmian47 | HZY |  |  |  |
| 313 | xuzhou142 | HZY | 338 | Deltapine-gy | USA |  |  |  |
| 314 | sumian9 | HZY | 339 | ji668 | HY |  |  |  |
| 315 | sumian12 | HZY | 340 | jimian25 | HY |  |  |  |
| 316 | sukang191 | HZY | 341 | jinmian5 | LN |  |  |  |
| 317 | gangmian1 | HZY | 342 | keke1543 | Others |  |  |  |
| 318 | gangmian2 | HZY | 343 | ningmian1 | HZY |  |  |  |
| 319 | kezi201 | USA | 344 | ningmian22 | HZY |  |  |  |
| 320 | daihongdai | HZY | 345 | nongda94-7 | HY |  |  |  |
| 321 | yishuhong | HZY | 346 | nongdamian8 | HY |  |  |  |
| 322 | bomian1 | HY | 347 | shumian1 | HZY |  |  |  |
| 323 | kemian4 | HZY | 348 | sumian1 | HZY |  |  |  |
| 324 | ganmian11 | HZY | 349 | sumian22 | HZY |  |  |  |
| 325 | ganmian12 | HZY | 350 | xiangmian10 | HZY |  |  |  |
