# Supplementary material for: Detection of Favorable QTL Alleles and Candidate Genes for Lint Percentage by GWAS in Chinese Upland Cotton
Source: Front Plant Sci. 2016 Oct 21;7:1576. doi: 10.3389/fpls.2016.01576 (PMC5073211; doi:10.3389/fpls.2016.01576)
Supplement: Supplementary Table S2 — Descriptive statistics for the lint percentage in the four different environments. [file Table2.DOCX]

Supplementary Table S2: Descriptive statistics for lint percentage in four different environments

| Environmentsa | Mean | SD | Min | Max | CV (%) | h^2^ (%) | G | G×E |
| --- | --- | --- | --- | --- | --- | --- | --- | --- |
| AY-14 | 41.05 | 2.87 | 29.46 | 48.13 | 7.00 | 69.72 | ** | *** |
| AY-15 | 39.70 | 3.18 | 26.58 | 47.22 | 8.02 |  |  |  |
| SHZ-14 | 41.78 | 2.78 | 28.58 | 46.93 | 6.66 |  |  |  |
| SHZ-15 | 41.58 | 2.64 | 29.32 | 47.61 | 6.34 |  |  |  |
| Mean | 41.03 | 2.65 | 28.49 | 46.17 | 6.47 |  |  |  |

^a^ AY-14, AY-15, SHZ-14 and SHZ-15: the different planting environments(AY: Anyang,Henan and SHZ: Shihezi, Xinjiang) in 2014 and 2015.

**,*** indicates significance at the 0.01 and 0.001 probability level, respectively.
