# Supplementary material for: Detection of Favorable QTL Alleles and Candidate Genes for Lint Percentage by GWAS in Chinese Upland Cotton
Source: Front Plant Sci. 2016 Oct 21;7:1576. doi: 10.3389/fpls.2016.01576 (PMC5073211; doi:10.3389/fpls.2016.01576)
Supplement: Supplementary Table S3 — Correlations between the different environments based on the 355 upland cotton accessions. [file Table3.DOCX]

Supplementary Table S3: Correlation coefficient of lint percentage among the four environments.

| Environments | AY-14 | AY-15 | SHZ-14 | SHZ-15 |
| --- | --- | --- | --- | --- |
| AY-14 | 1 | 0.7974^***^ | 0.8620^***^ | 0.7462^***^ |
| AY-15 |  | 1 | 0.8068^***^ | 0.7761^***^ |
| SHZ-14 |  |  | 1 | 0.8506^***^ |
| SHZ-15 |  |  |  | 1 |

^**^ significantly different (P < 0.001).
