# Supplementary material for: Detection of Favorable QTL Alleles and Candidate Genes for Lint Percentage by GWAS in Chinese Upland Cotton
Source: Front Plant Sci. 2016 Oct 21;7:1576. doi: 10.3389/fpls.2016.01576 (PMC5073211; doi:10.3389/fpls.2016.01576)
Supplement: Supplementary Table S4 — The 183 SSR primers whose sequences were obtained from public databases. [file Table4.DOCX]

| Supplementary Table S4 The 183 SSR primers whose sequences were obtained from public databases. |
| --- |
| \| N0. \| Primers \| N0. \| Primers \| N0. \| Primers \| N0. \| Primers \| N0. \| Primers \| \| --- \| --- \| --- \| --- \| --- \| --- \| --- \| --- \| --- \| --- \| \| 1 \| NAU3207 \| 41 \| BNL4108 \| 81 \| TMB1791 \| 121 \| NAU2451 \| 161 \| BNL1604 \| \| 2 \| NAU3260 \| 42 \| HAU0878 \| 82 \| NAU3778 \| 122 \| CIR183 \| 162 \| BNL3413 \| \| 3 \| HAU1888 \| 43 \| NAU2691 \| 83 \| NAU2909 \| 123 \| NAU2121 \| 163 \| NAU2933 \| \| 4 \| BNL2443 \| 44 \| HAU1022 \| 84 \| NAU2581 \| 124 \| JESPR65 \| 164 \| BNL3103 \| \| 5 \| NAU3012 \| 45 \| NAU3016 \| 85 \| CIR307 \| 125 \| JESPR101 \| 165 \| BNL3989 \| \| 6 \| PGML00820 \| 46 \| TMB0029 \| 86 \| Gh002 \| 126 \| NAU3110 \| 166 \| NAU5166 \| \| 7 \| NAU3053 \| 47 \| BNL2662 \| 87 \| HAU1455 \| 127 \| BNL4071 \| 167 \| DPL0443 \| \| 8 \| CIR219 \| 48 \| TMB0913 \| 88 \| Gh381 \| 128 \| BNL3279 \| 168 \| NAU2658 \| \| 9 \| HAU2662 \| 49 \| BNL1231 \| 89 \| CIR171 \| 129 \| NAU1042 \| 169 \| NAU4024 \| \| 10 \| NAU2251 \| 50 \| NAU3409 \| 90 \| DPL0742 \| 130 \| NAU1255 \| 170 \| NAU3308 \| \| 11 \| NAU6668 \| 51 \| Gh119 \| 91 \| Gh508 \| 131 \| TMB1268 \| 171 \| NAU3293 \| \| 12 \| BNL2652 \| 52 \| NAU6542 \| 92 \| NAU985 \| 132 \| TMB0409 \| 172 \| NAU3405 \| \| 13 \| NAU6664 \| 53 \| HAU0989 \| 93 \| PGML1273 \| 133 \| MUCS531 \| 173 \| BNL3474 \| \| 14 \| NAU5064 \| 54 \| NAU5013 \| 94 \| PGML01330 \| 134 \| BNL1665 \| 174 \| BNL1395 \| \| 15 \| NAU2811 \| 55 \| NAU2649 \| 95 \| NAU3995 \| 135 \| NAU4956 \| 175 \| TMB0471 \| \| 16 \| NAU3961 \| 56 \| NAU5164 \| 96 \| NAU1167 \| 136 \| Gh27 \| 176 \| NAU862 \| \| 17 \| BNL3452 \| 57 \| HAU2014 \| 97 \| CIR347 \| 137 \| NAU3017 \| 177 \| BNL2734 \| \| 18 \| BNL2705 \| 58 \| NAU3839 \| 98 \| NAU3828 \| 138 \| NAU6309 \| 178 \| BNL1394 \| \| 19 \| SWU0916 \| 59 \| HAU1300 \| 99 \| HAU1738 \| 139 \| NAU2926 \| 179 \| NAU1187 \| \| 20 \| BNL1672 \| 60 \| NAU3479 \| 100 \| JESPR204 \| 140 \| JESPR197 \| 180 \| NAU980 \| \| 21 \| HAU1693 \| 61 \| NAU2325 \| 101 \| CIR166 \| 141 \| NAU3519 \| 181 \| NAU3522 \| \| 22 \| NAU2508 \| 62 \| NAU868 \| 102 \| NAU5024 \| 142 \| NAU5408 \| 182 \| TMB1638 \| \| 23 \| NAU1274 \| 63 \| NAU922 \| 103 \| BNL1694 \| 143 \| NAU3414 \| 183 \| BNL1317 \| \| 24 \| NAU3639 \| 64 \| TMB0119 \| 104 \| PGML00353 \| 144 \| NAU2862 \|  \|  \| \| 25 \| NAU3714 \| 65 \| JESPR292 \| 105 \| NAU3917 \| 145 \| CIR406 \|  \|  \| \| 26 \| BNL252 \| 66 \| JESPR122 \| 106 \| NAU3385 \| 146 \| BNL3065 \|  \|  \| \| 27 \| BNL1521 \| 67 \| NAU3911 \| 107 \| BNL2895 \| 147 \| JESPR135 \|  \|  \| \| 28 \| NAU5444 \| 68 \| CM043 \| 108 \| NAU2742 \| 148 \| NAU2697 \|  \|  \| \| 29 \| BNL3590 \| 69 \| PGML00054 \| 109 \| BNL119 \| 149 \| HAU1185 \|  \|  \| \| 30 \| NAU2666 \| 70 \| HAU3236 \| 110 \| NAU2631 \| 150 \| MUSS167 \|  \|  \| \| 31 \| NAU1041 \| 71 \| HAU1617 \| 111 \| NAU6378 \| 151 \| NAU3273 \|  \|  \| \| 32 \| BNL3650 \| 72 \| BNL1705 \| 112 \| BNL4029 \| 152 \| TMB0206 \|  \|  \| \| 33 \| BNL2733 \| 73 \| BNL448 \| 113 \| JESPR220 \| 153 \| NAU2361 \|  \|  \| \| 34 \| BNL2872 \| 74 \| BNL3280 \| 114 \| BNL1404 \| 154 \| NAU6251 \|  \|  \| \| 35 \| BNL2961 \| 75 \| NAU3373 \| 115 \| NAU3269 \| 155 \| BNL3408 \|  \|  \| \| 36 \| NAU5152 \| 76 \| NAU3100 \| 116 \| NAU4034 \| 156 \| NAU3700 \|  \|  \| \| 37 \| NAU3398 \| 77 \| JESPR153 \| 117 \| BNL4030 \| 157 \| Gh485 \|  \|  \| \| 38 \| NAU5035 \| 78 \| NAU921 \| 118 \| BNL1440 \| 158 \| NAU6235 \|  \|  \| \| 39 \| HAU3050 \| 79 \| JESPR195 \| 119 \| NAU3206 \| 159 \| BNL2709 \|  \|  \| \| 40 \| PGML00802 \| 80 \| TMB0366 \| 120 \| DPL0279 \| 160 \| NAU3325 \|  \|  \| |
