# Supplementary material for: Detection of Favorable QTL Alleles and Candidate Genes for Lint Percentage by GWAS in Chinese Upland Cotton
Source: Front Plant Sci. 2016 Oct 21;7:1576. doi: 10.3389/fpls.2016.01576 (PMC5073211; doi:10.3389/fpls.2016.01576)
Supplement: Supplementary Table S5 — The 98 SSR primers whose sequences were not obtained from public databases. [file Table5.DOCX]

| Supplementary Table S5 The 98 SSR primers whose sequences were not obtained from public databases s |
| --- |
| \| N0. \| Primers \| N0. \| Primers \| N0. \| Primers \| N0. \| Primers \| N0. \| Primers \| \| --- \| --- \| --- \| --- \| --- \| --- \| --- \| --- \| --- \| --- \| \| 1 \| ACT/CAC3 \| 21 \| DPL0582 \| 41 \| dPL0281 \| 61 \| P1 \| 81 \| M7E7 \| \| 2 \| MGHES55 \| 22 \| NBRI1837 \| 42 \| NAU855 \| 62 \| GML00720 \| 82 \| M6E8 \| \| 3 \| MGHES31 \| 23 \| Lc1 \| 43 \| JESPR502 \| 63 \| SHIN-1452 \| 83 \| T28E7 \| \| 4 \| MGHES46 \| 24 \| CGR5707 \| 44 \| cgr5161 \| 64 \| CGR5621 \| 84 \| TMHA73 \| \| 5 \| MGHES16 \| 25 \| Lg \| 45 \| dPL0044 \| 65 \| HAU916 \| 85 \| M8E17 \| \| 6 \| MGHES63 \| 26 \| N1 \| 46 \| NAU462 \| 66 \| GML00802 \| 86 \| NAU6094 \| \| 7 \| MGHES66 \| 27 \| DPL0095 \| 47 \| M8E2 \| 67 \| CGR6528 \| 87 \| NAU1453 \| \| 8 \| T1 \| 28 \| CER0028 \| 48 \| E6M6 \| 68 \| TMHA20-A17 \| 88 \| TML04 \| \| 9 \| NAU1587 \| 29 \| CGR6880 \| 49 \| M9E16 \| 69 \| TMK19 \| 89 \| T43E10 \| \| 10 \| CGR6733 \| 30 \| CGR5452 \| 50 \| M7E2 \| 70 \| DPL5132 \| 90 \| M4E10 \| \| 11 \| HAU0215 \| 31 \| MGHES44 \| 51 \| T15E16 \| 71 \| AGG/CTC1 \| 91 \| E7M6 \| \| 12 \| DC40188 \| 32 \| NAU7655 \| 52 \| M2E13 \| 72 \| BNL3154 \| 92 \| T45E13 \| \| 13 \| DPL0400 \| 33 \| dPL0864 \| 53 \| T5E3 \| 73 \| TML21 \| 93 \| E19M5 \| \| 14 \| CGR5581 \| 34 \| dPL0622 \| 54 \| JESP261 \| 74 \| TMD05 \| 94 \| NAU1595 \| \| 15 \| DC40217 \| 35 \| dc40182 \| 55 \| T44E11 \| 75 \| JNAU3201 \| 95 \| CGR5001 \| \| 16 \| CGR5018 \| 36 \| dPL0391 \| 56 \| JESP134 \| 76 \| CGR5732 \| 96 \| CER0152 \| \| 17 \| CGR6680 \| 37 \| Gh4282 \| 57 \| dPL0170 \| 77 \| DPL0491 \| 97 \| HAU329 \| \| 18 \| DPL0492 \| 38 \| dPL0504 \| 58 \| GhPEL \| 78 \| CGR6930 \| 98 \| NAU1590 \| \| 19 \| PGML04142 \| 39 \| NAU2540 \| 59 \| im \| 79 \| CGR5590 \|  \|  \| \| 20 \| CGR6185 \| 40 \| cgr5800 \| 60 \| PGML03773 \| 80 \| TMO05 \|  \|  \| |
