# Supplementary material for: Detection of Favorable QTL Alleles and Candidate Genes for Lint Percentage by GWAS in Chinese Upland Cotton
Source: Front Plant Sci. 2016 Oct 21;7:1576. doi: 10.3389/fpls.2016.01576 (PMC5073211; doi:10.3389/fpls.2016.01576)
Supplement: Supplementary Table S6 — The QTLs of FL and FS from 23 reports of QTL mapping. [file Table6.DOCX]

| Supplementary Table S6 The QTLs of FL and FS from 23 reports of QTL mapping. |
| --- |
| \| No. of reference \| QTLs \| marker1 \| marker2 \| Nearest Marker \| Chro. \| \| --- \| --- \| --- \| --- \| --- \| --- \| \| 1 \| LP1 \| BNL2662 \| BNL3279 \|  \| 5 \| \| LP2 \| BNL4108 \| BNL4030 \|  \| 6 \| \| LP3 \| BNL3280 \| AGG/CTC1 \|  \| 18/20 \| \| LP4 \| ACT/CAC3 \| BNL2895 \|  \| Uncertain \| \| 2 \| 1 \| MGHES55 \|  \|  \|  \| \| 2 \| TMB0366 \|  \|  \|  \| \| 3 \| TMB0409 \|  \|  \|  \| \| 4 \| MGHES31 \|  \|  \|  \| \| 5 \| TMB0119 \|  \|  \|  \| \| 6 \| JESPR153 \|  \|  \|  \| \| 7 \| TMB0471 \|  \|  \|  \| \| 8 \| TMB0029 \|  \|  \|  \| \| 9 \| MGHES46 \|  \|  \|  \| \| 10 \| JESPR65 \|  \|  \|  \| \| 11 \| JESPR122 \|  \|  \|  \| \| 12 \| MGHES16 \|  \|  \|  \| \| 13 \| MGHES63 \|  \|  \|  \| \| 14 \| TMB0206 \|  \|  \|  \| \| 15 \| MGHES66 \|  \|  \|  \| \| 16 \| JESPR292 \|  \|  \|  \| \| 17 \| JESPR220 \|  \|  \|  \| \| 3 \| 04Lint \| BNL3650 \|  \|  \| c6 \| \| 05Lint \| T1 \|  \|  \|  \| \| 05Lint \| BNL4108 \|  \|  \|  \| \| 4 \| qLP-D2-1 \| NAU3308 \|  \|  \| D2 \| \| qLP-A10-1 \| NAU2451 \|  \|  \| A10 \| \| 5 \| qLP-D8-1 \| NAU1587 \| BNL3154 \|  \| D8 \| \|  \| NAU1587 \| NAU3207 \|  \| D8 \| \|  \| BNL3474 \| TML21 \|  \| D8 \| \| qLP-D8-2 \| BNL1521 \| TMD05 \|  \| D8 \| \|  \| BNL1521 \| JNAU3201 \|  \| D8 \| \| qLP-D8-3 \| NAU2926 \| TML21 \|  \| D8 \| \|  \| BNL3474 \| NAU3207 \|  \| D8 \| \|  \| BNL3474 \| TML21 \|  \| D8 \| \| 6 \| qLP‐C5‐1 \| SWU0916 \|  \|  \| c5 \| \| qLP‐C5‐2 \| CGR6733 \|  \|  \| c5 \| \| qLP‐C5‐3 \| HAU0215 \|  \|  \| c5 \| \| qLP‐C9‐1 \| HAU1617 \|  \|  \| c9 \| \| qLP‐C10‐1 \| BNL1665 \|  \|  \| c10 \| \| qLP‐C10‐2 \| DC40188 \|  \|  \| c10 \| \| qLP‐C12‐1 \| DPL0400 \|  \|  \| c12 \| \| qLP‐C12‐2 \| DPL0443 \|  \|  \| c12 \| \| qLP‐C14‐1 \| CGR5581 \|  \|  \| c14 \| \| qLP‐C14‐2 \| HAU1888 \|  \|  \| c14 \| \| qLP‐C15‐1 \| HAU3050 \|  \|  \| c15 \| \|  \| DC40217 \|  \|  \| c15 \| \| qLP‐C15‐2 \| NAU3714 \|  \|  \| c15 \| \| qLP‐C16‐1 \| PGML01330 \|  \|  \| c16 \| \| qLP‐C16‐2 \| NAU6664 \|  \|  \| c16 \| \| qLP‐C16‐3 \| Gh002 \|  \|  \| c16 \| \| qLP‐C16‐4 \| PGML00820 \|  \|  \| c16 \| \| qLP‐C16‐5 \| CGR5018 \|  \|  \| c16 \| \| qLP‐C16‐6 \| BNL3065 \|  \|  \| c16 \| \| qLP‐C16‐7 \| CGR6680 \|  \|  \| c16 \| \| qLP‐C16‐8 \| DPL0492 \|  \|  \| c16 \| \| qLP‐C17‐1 \| NAU6542 \|  \|  \| c17 \| \| qLP‐C17‐2 \| JESPR195 \|  \|  \| c17 \| \| qLP‐C17‐3 \| DPL0279 \|  \|  \| c17 \| \| qLP‐C17‐4 \| PGML04142 \|  \|  \| c17 \| \| qLP‐C17‐5 \| CGR6185 \|  \|  \| c17 \| \| qLP‐C21‐1 \| DPL0582 \|  \|  \| c21 \| \| 7 \| qLP01.1 \| PGML1273 \|  \|  \| Chr01 \| \| qLP17.1 \| NAU2649 \|  \|  \| Chr17 \| \| Chr17 \| NBRI1837 \|  \|  \| Chr17 \| \| qLP06.1 \| T1 \|  \|  \| Chr06 \| \| qLP07.1 \| Lc1 \|  \|  \| Chr07 \| \| qLP09.1 \| CGR5707 \|  \|  \| Chr09 \| \| qLP21.1 \| Lg \|  \|  \| Chr21 \| \| qLP12.1 \| N1 \|  \|  \| Chr12 \| \| qLP26.1 \| NAU5164 \|  \|  \| Chr26 \| \| Chr26 \| HAU1738 \|  \|  \| Chr26 \| \| 8 \| qLP_3a(F2) \| NAU2742 \| NAU1167 \|  \| c3 \| \| qLP_3b(F2) \| DPL0095 \| NAU3839 \|  \| c3 \| \| qLP_3(F2:3) \| DPL0095 \| NAU3839 \|  \| c3 \| \| qLP_3c(F2) \| CER0028 \| HAU1455 \|  \| c3 \| \| qLP_19a(F2) \| NAU1042 \| CGR5732 \|  \| c19 \| \| qLP_19b(F2) \| NAU1255 \| HAU0878 \|  \| c19 \| \| qLP_19(F2:3) \| NAU1255 \| HAU0878 \|  \| c19 \| \| qLP_26a(F2) \| CGR6880 \| DPL0491 \|  \| c26 \| \| qLP_26b(F2) \| CGR5452 \| CGR6930 \|  \| c26 \| \| qLP_26(F2:3) \| MGHES44 \| DPL0742 \|  \| c26 \| \| 9 \| qLP-08A-c5-1 \| NAU4034 \| NAU3405 \|  \| c5 \| \| qLP-08A-c7-1 \| BNL1694 \| BNL1604 \|  \| c7 \| \| qLP-08A-c16-1 \| BNL2734 \| NAU5024 \|  \| c16 \| \| qLP-08A-c16-2 \| NAU5024 \| BNL1694 \|  \| c16 \| \| qLP-07X-c7-1 \| NAU5152 \| BNL2733 \|  \| c7 \| \| 10 \|  \| NAU1041 \|  \|  \| A10 \| \|  \| NAU2631 \|  \|  \| A10 \| \|  \| NAU7655 \|  \|  \| A10 \| \|  \| NAU2933 \|  \|  \| A11 \| \|  \| NAU1274 \|  \|  \| A12 \| \|  \| BNL2709 \|  \|  \| A12 \| \|  \| dPL0864 \|  \|  \| A13 \| \|  \| NAU3017 \|  \|  \| A13 \| \|  \| NAU3373 \|  \|  \| A3 \| \|  \| BNL3279 \|  \|  \| A3 \| \|  \| dPL0622 \|  \|  \| A5 \| \|  \| dc40182 \|  \|  \| A7 \| \|  \| Gh27 \|  \|  \| A9 \| \|  \| NAU3414 \|  \|  \| A9 \| \|  \| dPL0391 \|  \|  \| D1 \| \|  \| Gh4282 \|  \|  \| D10 \| \|  \| BNL119 \|  \|  \| D10 \| \|  \| dPL0504 \|  \|  \| D10 \| \|  \| NAU2540 \|  \|  \| D10 \| \|  \| cgr5800 \|  \|  \| D11 \| \|  \| HAU3236 \|  \|  \| D2 \| \|  \| dPL0281 \|  \|  \| D3 \| \|  \| NAU2691 \|  \|  \| D3 \| \|  \| NAU855 \|  \|  \| D3 \| \|  \| NAU868 \|  \|  \| D3 \| \|  \| JESPR502 \|  \|  \| D4 \| \|  \| NAU3110 \|  \|  \| D5 \| \|  \| NAU3911 \|  \|  \| D7 \| \|  \| HAU2662 \|  \|  \| D7 \| \|  \| cgr5161 \|  \|  \| D8 \| \|  \| dPL0044 \|  \|  \| D9 \| \|  \| NAU462 \|  \|  \| D9 \| \|  \| BNL1317 \|  \|  \| D9 \| \| 11 \| qLP-3 \| DPL0095 \| NAU3839 \|  \| c3 \| \| qLP-19 \| NAU1187 \| NAU1255 \|  \| c19 \| \| qLP-26 \| MGHES44 \| DPL0742 \|  \| c26 \| \| qLP-3 \| DPL0095 \| NAU3839 \|  \| c3 \| \| qLP-5/19 \| NAU2811 \| CGR5590 \|  \| c5 \| \| qLP-19 \| BNL3452 \| NAU3012 \|  \| c19 \| \| 12 \| F2:3-qLP-c10-1 \| NAU921 \| TMO05 \| T43E10 \| c10 \| \| F2:3-qLP-c10-2 \| M8E2 \| BNL2705 \| BNL2872 \| c10 \| \| F2:3-qLP-c10-3 \| E6M6 \| M7E7 \| CIR166 \| c10 \| \| F2:3-qLP-c25-1 \| BNL1440 \| BNL1440 \| BNL3103 \| c25 \| \| F2:3-qLP-c13-1 \| M9E16 \| M9E16 \| TMHA73 \| c13 \| \| F2:3-qLP-c13-2 \| M7E2 \| M6E8 \| BNL3989 \| c13 \| \| F2:3-qLP-c13-3 \| T15E16 \| T28E7 \| M4E10 \| c13 \| \| TC-qLP-c2-1 \| M2E13 \| M2E13 \| BNL3590 \| c2 \| \| TC-qLP-c2-2 \| BNL3413 \| BNL3413 \| E7M6 \| c2 \| \| TC-qLP-c5-1 \| T5E3 \| NAU922 \| BNL4071 \| c5 \| \| TC-qLP-c10-1 \| JESP261 \| CIR171 \| CIR171 \| c10 \| \| TC-qLP-c10-2 \| NAU921 \| TMO05 \| T43E10 \| c10 \| \| TC-qLP-c13-1 \| T44E11 \| TMHA73 \| BNL4029 \| c13 \| \| TC-qLP-c13-2 \| CIR406 \| CIR406 \| BNL1394 \| c13 \| \| TC-qLP-c17-1 \| BNL3408 \| BNL3408 \| BNL3408 \| c17 \| \| TC-qLP-c18-1 \| JESP134 \| M8E17 \| BNL2652 \|  \| \| TC-qLP-c22-1 \| CIR183 \| CIR183 \| T45E13 \|  \| \| 13 \| qLP-A1-1 \| NAU6251 \| NAU6309 \|  \| A1 \| \| qlp-A5-2 \| NAU2121 \| NAU6094 \|  \| A5 \| \| qLP-A9-1 \| NAU2666 \| NAU6668 \|  \| A9 \| \| Qlp-D9-1 \| NAU2658 \| BNL1672 \|  \| D9 \| \| Qlp-A11-2 \| NAU5064 \| NAU1453 \|  \| A11 \| \| 14 \| qLp-A-1 \| dPL0170 \| NAU3995 \|  \| A2 \| \| qLp-B-1 \| NAU3639 \| im \|  \| A2 \| \| qLp-C-1 \| NAU5035 \| TML04 \|  \| A2 \| \| qLp-C-2 \| GhPEL \| HAU1022 \|  \| A2 \| \| qLp-C-3 \| BNL2443 \| cgr6528 \|  \| A2 \| \| qLp-C-4 \| NAU3479 \| NAU3016 \|  \| A2 \| \| qLp-D-1 \| im \| NAU5444 \|  \| A2 \| \| qLp-E-1 \| im \| NAU3479 \|  \| A2 \| \| 15 \| qLP-3-12 \| HAU1022 \|  \|  \| c3 \| \|  \| HAU1022 \|  \|  \| c3 \| \|  \| HAU1022 \|  \|  \| c3 \| \| qLP-11-11 \| NAU3409 \|  \|  \| c11 \| \| qLP-12-13 \| HAU0989 \|  \|  \| c12 \| \|  \| HAU0989 \|  \|  \| c12 \| \| qLP-13-5 \| PGML03773 \|  \|  \| c13 \| \| qLP-17-5 \| HAU2014 \|  \|  \| c17 \| \| qLP-17-7 \| NAU2909 \|  \|  \| c17 \| \|  \| NAU2909 \|  \|  \| c17 \| \|  \| NAU2909 \|  \|  \| c17 \| \| qLP-17-8 \| NAU2325 \|  \|  \| c17 \| \|  \| NAU2325 \|  \|  \| c17 \| \|  \| NAU2325 \|  \|  \| c17 \| \| qLP-20-9 \| NAU5013 \|  \|  \| c20 \| \| qLP-20-11 \| Gh119 \|  \|  \| c20 \| \| 16 \| qLP-A11-1 \| BNL1231 \| E19M5 \|  \| A11 \| \| qLP-A5-1 \| BNL3452 \| NAU3828 \|  \| A5 \| \| qLP-A5-2 \| P1 \| NAU3273 \|  \| A5 \| \| qLP-A10-1 \| NAU3260 \| NAU1595 \|  \| A10 \| \| 17 \| qLP-2-1 \| GML00720 \| NAU6378 \|  \| c2 \| \| qLP-2-2 \| SHIN-1452 \| PGML00353 \|  \| c2 \| \| qLP-4-1 \| CGR5621 \| NAU4956 \|  \| c4 \| \| qLP-4-2 \| PGML00054 \| HAU916 \|  \| c4 \| \| qLP-4-3 \| HAU916 \| NAU5408 \|  \| c4 \| \| qLP-6-1 \| HAU1693 \| PGML00802 \|  \| c6 \| \| qLP-6-2 \| GML00802 \| CGR5001 \|  \| c6 \| \| qLP-16-1 \| NAU6235 \| CER0152 \|  \| c16 \| \| qLP-17-1 \| HAU1300 \| HAU329 \|  \| c17 \| \| 18 \|  \| CIR219 \| NAU1590 \|  \| Ch r3 \| \|  \| BNL2961 \| NAU985 \|  \| LGD03 \| \| 19 \| qLP-3-15.6 \| CGR6528 \| CIR347 \|  \| LG03 \| \| qLP-16-0.0 \| CM043 \| BNL3474 \|  \| LG16 \| \| qLP-2-89.3 \| TMHA20-A17 \| TMB0913 \|  \| LG02 \| \| qLP-3-18.6 \| CIR347 \| CGR6528 \|  \| LG03 \| \| qLP-5-17.8 \| MUSS167 \| MUCS531 \|  \| LG05 \| \| qLP-22-4 \| Gh381 \| Gh485 \|  \| LG22 \| \| 20 \|  \| CIR307 \|  \|  \| D1 \| \|  \| BNL3590 \|  \|  \| A2 \| \|  \| JESPR197 \|  \|  \| A5 \| \|  \| NAU2581 \|  \|  \| UL \| \|  \| NAU3053 \|  \|  \| D7 \| \|  \| NAU3206 \|  \|  \| A6 \| \|  \| NAU3293 \|  \|  \| D12 \| \|  \| NAU3308 \|  \|  \| D2 \| \|  \| NAU3522 \|  \|  \| A13 \| \|  \| NAU3778 \|  \|  \| A12 \| \|  \| NAU3995 \|  \|  \| A3 \| \|  \| BNL1395 \|  \|  \| D7 \| \|  \| JESPR204 \|  \|  \| D13 \| \|  \| NAU862 \|  \|  \| A3 \| \|  \| BNL1672 \|  \|  \| A9 \| \|  \| BNL1705 \|  \|  \| D11 \| \|  \| NAU2251 \|  \|  \| A12 \| \| 21 \|  \| NAU3269 \|  \|  \| A05(Chr.05) \| \|  \| NAU5166 \|  \|  \| A10(Chr.10) \| \|  \| NAU2508 \|  \|  \| A10(Chr.10) \| \|  \| NAU980 \|  \|  \| A11(Chr.11) \| \|  \| JESPR135 \|  \|  \| A11(Chr.11) \| \|  \| NAU3398 \|  \|  \| A13(Chr.13) \| \|  \| JESPR204 \|  \|  \| A13(Chr.13) \| \|  \| BNL3590 \|  \|  \| D03(Chr.17) \| \|  \| TMK19 \|  \|  \| D06(Chr.25) \| \|  \| NAU3100 \|  \|  \| D09(Chr.23) \| \|  \| NAU3917 \|  \|  \| D10(Chr.20) \| \|  \| BNL1404 \|  \|  \| D11(Chr.21) \| \|  \| Gh508 \|  \|  \| D11(Chr.21) \| \|  \| NAU2361 \|  \|  \| D11(Chr.21) \| \| 22 \|  \| NAU3385 \|  \|  \| A1(Chr.1) \| \|  \| JESPR101 \|  \|  \| A2(Chr.2) \| \|  \| NAU5166 \|  \|  \| A10(Chr.10) \| \|  \| NAU4024 \|  \|  \| D2(Chr.14) \| \|  \| NAU3700 \|  \|  \| D3(Chr.17) \| \|  \| BNL448 \|  \|  \| D4(Chr.22) \| \|  \| NAU1042 \|  \|  \| D5(Chr.19) \| \|  \| BNL3103 \|  \|  \| D6(Chr.25) \| \|  \| NAU4956 \|  \|  \| D7(Chr.16) \| \|  \| BNL252 \|  \|  \| D8(Chr.24) \| \|  \| NAU3414 \|  \|  \| D9(Chr.23) \| \|  \| NAU3961 \|  \|  \| D12(Chr.26) \| \|  \| NAU2697 \|  \|  \| D13(Chr.18) \| \| 23 \|  \| NAU3325 \|  \|  \|  \| \|  \| NAU3519 \|  \|  \|  \| \|  \| HAU1185 \|  \|  \|  \| \|  \| TMB1638 \|  \|  \|  \| \|  \| NAU3110 \|  \|  \|  \| \|  \| JESPR101 \|  \|  \|  \| \|  \| TMB1268 \|  \|  \|  \| \|  \| TMB1791 \|  \|  \|  \| \|  \| DPL5132 \|  \|  \|  \| \|  \| NAU2862 \|  \|  \|  \| \|  \| BNL1231 \|  \|  \|  \| |
| 1. Zhang et al. Construction of a genetic linkage map and QTL analysis of fiber-related traits in upland cotton (*Gossypium hirsutum* L.) *Euphytica* (2005) 144: 91-99.  2. Abdurakhmonov et al. Microsatellite markers associated with lint percentage traitin cotton, *Gossypium hirsutum*. *Euphytica* (2007) 156:141-156.  3. Wan et al.T1 locus in cotton is the candidate gene affecting lint percentage, fiber quality and spiny bollworm (Earias spp.) resistance. *Euphytica* (2007) 158:241-247.  4. Qin et al.QTL mapping of yield and fiber traits based on a four-way cross population in *Gossypium hirsutum* L. *Theor Appl Genet* (2008) 117:883-894.  5. Chen et al. Using three selected overlapping RILs to fine-map the yield component QTL on Chro.D8 in Upland cotton. *Euphytica* (2010) 176:321-329  6. Shi et al.Constructing a high‐density linkage map for *Gossypium hirsutum* × *Gossypium barbadense* and identifying QTLs for lint percentage. *J Integr Plant Biol* (2015) 57, 450-467.  7. Liu et al. Construction of a high‑density genetic map and lint percentage and cottonseed nutrient trait QTL identification in upland cotton (*Gossypium hirsutum* L.). *Mol Genet Genomics* (2015) 290:1683-1700.  8. Xia, et al. Major gene identification and quantitative trait locus mapping for yield-related traits in upland cotton (*Gossypium hirsutum* L.) *Journal of Integrative Agriculture* (2014). 13, 299-309.  9. Yu et al.Mapping quantitative trait loci for lint yield and fiber quality across environments in a *Gossypium hirsutum* × *Gossypium barbadense* backcross inbred line population *Theor Appl Genet* (2013b) 126:275-287.  10. Zhang et al. New QTLs for lint percentage and boll weight mined in introgression lines from two feral landraces into *Gossypium hirsutum* acc TM-1 *Plant Breeding* (2016) 135, 90-101.  11. Wang et al. Quantitative trait loci mapping and genetic dissection for lint percentage in upland cotton (*Gossypium hirsutum*). *J. Genet.* (2014) 93, 371-378.  12. Yu, et al. Identification of quantitative trait loci across interspecific F_2_, F_2:3_ and testcross populations for agronomic and fiber traits in tetraploid cotton. *Euphytica* (2013a) 191, 375-389.  13. Liu et al.Quantitative trait loci mapping for yield and its components by using two immortalized populations of a heterotic hybrid in *Gossypium hirsutum* L. *Mol Breeding* (2012) 29:297-311.  14. Wang, et al. The im mutant gene negatively affects many aspects of fiber quality traits and lint percentage in cotton. *Crop Sci*. (2013) 52, 27-37.  15. Zhai, et al. Identification of chromosome segment substitution lines of *Gossypium barbadense* introgressed in *G. hirsutum* and quantitative trait locus mapping for fiber quality and yield traits. *PLoS One* (2016),11, e0159101.  16.Wang et al. QTL mapping of yield and yield components for elite hybrid derived-rils in upland cotton. *Journal of Genetics and Genomics* (2007) 34, 35-45.  17. Jia et al. Identification of qtl for boll weight and lint percentage of upland cotton (*Gossypium hirsutum* L.) RIL population in multiple environments. *Molecular Plant Breeding* (2011) 9, 318-326.  18. Zhang et al. Genetic analysis and QTL stagging of lint percentage and its closely related yield components in upland cotton. *Jiang su J. of Agr. Sci*. (2005) 21,264-271.  19. Zhang, et al. QTL analysis on yield and its components in upland cotton RIL. *Acta Agron. Sin*. (2011) 37, 433-442.  20. Qin, et al. Identification of associated SSR markers for yield component and fiber quality traits based on frame map and upland cotton collections. *PLoS One* (2015) 10, e0118073.  21. Mei, et al. Favorable QTL alleles for yield and its components identified by association mapping in Chinese upland cotton cultivars. *PLoS One* (2013) 8, e82193.  22. Zhang et al. Variations and transmission of qtl alleles for yield and fiber qualities in upland cotton cultivars developed in China. *PLoS One* (2013) 8, e57220.  23. Jia et al. Association mapping for epistasis and environmental interaction of yield traits in 323 cotton cultivars under 9 different environments. *PLoS One* (2014) 9, e95882. |
